# Nutrient–Nutrient Interactions Among Broccoli Glucosinolates and Their Implications for Breeding Cruciferous Crops to Enhance Human Health

**DOI:** 10.3390/nu17020344

**Published:** 2025-01-18

**Authors:** Weston Bussler, Katelyn DeZego, Holli Chandler, Robert W. Reid, Slavko Komarnytsky

**Affiliations:** 1 Plants for Human Health Institute, North Carolina State University, 600 Laureate Way, Kannapolis, NC 28081, USA; 2 Department of Food, Bioprocessing, and Nutrition Sciences, North Carolina State University, 400 Dan Allen Drive, Raleigh, NC 27695, USA; 3 Department of Biology, Catawba College, 2300 W Innes St., Salisbury, NC 28144, USA; 4 Department of Bioinformatics and Genomics, University of North Carolina at Charlotte, 150 N Research Campus Dr, Kannapolis, NC 28081, USA; rreid2@charlotte.edu

**Keywords:** whole foods, vegetables, breeding, nutrient interactions, glucosinolate profile, mixture analysis, cell proliferation, chemoprevention, colon

## Abstract

While a balanced diet can fulfill most nutritional needs, optimizing the composition of specific foods like broccoli can amplify their health benefits. Background/Objectives: Broccoli (*Brassica oleracea* L. Italica group) is a widely consumed cruciferous vegetable valued for its gastrointestinal and immune health benefits. However, the individual contributions and interactions of broccoli glucosinolates, as they hydrolyze into bioactive isothiocyanates, remain poorly understood. Methods: This study investigated mixtures of four major aliphatic glucosinolates—glucoraphanin, gluconapin, progoitrin, and sinigrin—in individual and combinational models to assess their effects on human colorectal cell proliferation. Results: Combination index analysis revealed moderate to strong antagonistic interactions among these glucosinolates, with the most significant antagonism observed during enzymatic hydrolysis by myrosinase. Mixture analysis identified an optimal glucosinolate ratio including glucoraphanin (81–84%), gluconapin (9–19%), and others (0–7%) to maximize their antiproliferative effects (adjusted R^2^ > 0.80). This optimal profile was achievable within the target broccoli mapping population. Testing the near-optimal VB067 isogenic broccoli line showed a 44% increase in antiproliferative activity compared to the initial breeding parent or an average sister line. Conclusions: This study highlights the potential of leveraging nutrient–nutrient interactions to guide molecular breeding and produce functional varieties of cruciferous vegetables with optimized health benefits.

## 1. Introduction

Whole foods, such as fruits, vegetables, whole grains, and nuts, are nutrient-dense and support overall health by retaining the natural nutrients, fiber, and phytochemicals often lost during processing [1,2]. Their fiber and phytochemicals promote gut health by nourishing beneficial bacteria, enhancing digestion, and boosting immunity [3]. Traditionally, studying phytochemical groups based on their chemical structures and biological activities, rather than individual compounds, highlighted their collective roles in supporting human health [4].

As part of a whole-foods diet, broccoli (*Brassica oleracea* var. *italica* Plenck, family Brassicaceae) provides an unprocessed option that supports gastrointestinal and immune health [5]. Diets emphasizing plant-based foods like broccoli are associated with improved longevity and reduced cancer risk, including colorectal cancer—the second leading cause of cancer deaths in the United States [6,7]. Epidemiological studies and recent meta-analyses consistently show an inverse correlation between broccoli consumption and colorectal cancer risk [8,9,10].

**Figure 1:**
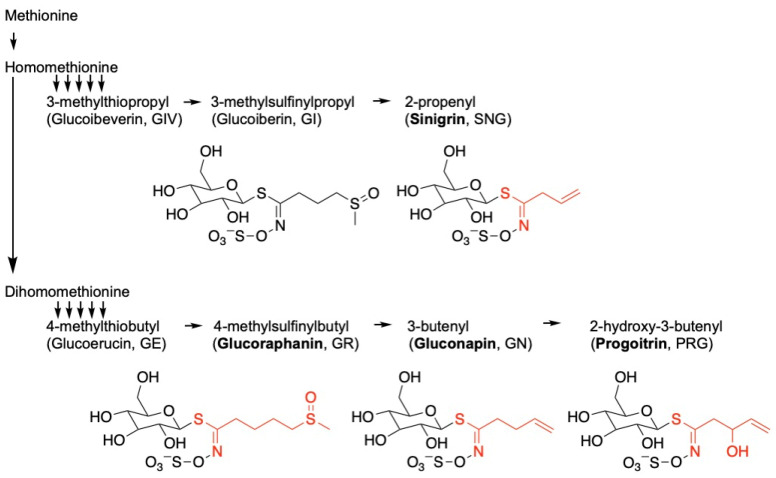
Schematic pathway and structural relationships among the aliphatic glucosinolates. The compounds used in this study are in bold, and their respective isothiocyanates are in red. There are multiple enzymatic steps between homomethionine or dihomomethionine and the respective metabolites (abbreviated as multiple arrows).

Glucosinolates are a major class of sulfur-containing secondary metabolites commonly found in broccoli and other cruciferous vegetables [11]. Naturally occurring glucosinolates are classified as aliphatic, aromatic, or indole depending on their amino acid precursors [12]. Those derived from alanine, leucine, isoleucine, valine, and methionine are categorized as aliphatic [13]. The most common broccoli glucosinolates are glucoraphanin (GR), gluconapin (GN), progoitrin (PG), glucoiberin (GIB), sinigrin (SGN), glucobrassicin (GB), neoglucobrassicin (NGB), and gluconasturitiin (GNT) (Figure 1), with the first five being the major aliphatic glucosinolates in this crop [14]. Glucosinolates alone lack significant chemopreventive activity [15]; however, their enzymatic breakdown by plant or microbial myrosinase (thioglucoside glucohydrolase, EC 3.2.1.147) following plant tissue damage produces isothiocyanates [16,17]. These metabolites are primarily associated with chemopreventive and other health benefits observed in humans [18,19]. Multiple methods of action have been suggested for isothiocyanate bioactivity, including modulating the activity of phase I and phase II detoxification enzymes [20], activating the NRF2/ARE pathway [21], inducing cell the cycle arrest and apoptosis of cancerous cells [22], and inhibiting histone deacetylases [23].

Studies investigating total glucosinolate content in various broccoli cultivars revealed that genetics, environmental conditions, and post-harvest processing all significantly influence both the quantity and chemical composition of individual glucosinolates [24,25,26]. Initial efforts to increase total glucosinolates in broccoli have been made by increasing sulfur content in soil [27]; however, this strategy did not address the differences in biological activity, individual contributions, and interactions among different glucosinolates. To specifically enhance the chemopreventive properties of broccoli, recent breeding programs have increasingly focused on optimizing levels of secondary metabolites, particularly glucosinolates and their hydrolysis products [28,29,30]. This shift partially countered traditional breeding priorities, which emphasized reducing bitter phytochemicals [31] and maximizing yield, pest resistance, and shelf-life, often at the expense of nutritional and health benefits [32].

Glucoraphanin (GR), the precursor to isothiocyanate sulforaphane, is the best characterized glucosinolate in broccoli that mediates its chemopreventative properties [33]. Previous targeted approaches to increase broccoli glucoraphanin by crossbreeding it with wild relatives such as *Brassica villosa* Biv. resulted in significant changes in individual glucosinolate profiles together with the often undesirable shifts in agricultural parameters and functionality of the crop [28,29]. A more recently constructed high-density genetic linkage map based on the F2:3 population of two broccoli accessions offered a unique opportunity for developing novel targeted breeding programs focused on plants with optimized glucosinolate profiles [30]. Moreover, significant differences in biological activity and enzymatic hydrolysis by myrosinase within the food matrix among individual glucosinolates make the conventional “single nutrient” approach to their evaluation unsatisfactory. Despite the previous development of the combination index (CI)-isobologram equation to identify additive, antagonistic, or synergistic interactions [34], it is often insufficient for capturing the dynamic interactions between compounds.

The primary objective of this study was therefore to evaluate the antiproliferative effects of closely related aliphatic glucosinolates in broccoli, focusing on their interactions during hydrolysis into bioactive isothiocyanates, and to identify a subset of glucosinolates critical for maximizing the target biological activity of broccoli cultivars. To make the first step in addressing these challenges, we have designed a proof-of-concept study that examined antiproliferative contributions of individual broccoli aliphatic glucosinolates and their mixtures when activated at the level of myrosinase, and prior to challenging the HT-29 human colorectal adenocarcinoma cells. We then used a mixture analysis commonly applied in food science to evaluate food ingredient functionality or consumer desirability [35,36] to obtain a predictive model of their combined antiproliferative effects and determine the optimal ratios to maximize the antiproliferative potential of broccoli crops. This approach provides an additional tool for breeding programs to optimize phytochemical profiles in broccoli, aiming to enhance its antiproliferative potential in support of gastrointestinal and immune health.

## 2. Materials and Methods

### 2.1. Broccoli Mapping Population

The F2:3 broccoli mapping population was established previously as a cross between two broccoli accessions, a calabrese-type double haploid VI-158 and a brocolette neri-type cultivar BNC (USDA PI 462209) [26]. The 136 and 146 F2:3 broccoli families were grown at two different location over two growing seasons, and their glucosinolate profiles have been evaluated by HPLC [30]. This material was kindly provided by Dr. Allan F. Brown (currently at International Institute of Tropical Agriculture, Arusha, Tanzania) and subsequently screened to identify broccoli isogenic lines with a near-optimal ratio of aliphatic glucosinolates based on their highest antiproliferative effects against human colorectal adenocarcinoma cells as described below.

### 2.2. Chemicals and Reagents

All chemicals and solvents (anhydrous and ACS grade) were purchased from Sigma-Aldrich (St. Louis, MO, USA) unless specified otherwise. Aliphatic glucosinolate standards of glucoraphanin, gluconapin, progoitrin, and sinigrin were purchased from Chromodex (Irvine, CA, USA).

### 2.3. Myrosinase Treatments

Myrosinase (thioglucoside glucohydrolase, EC 3.2.1.147) isolated from *Sinapis alba* L. (white mustard) seeds was purchased from Sigma-Aldrich (St. Louis, MO, USA), aliquoted in 2.5 U/mL stocks, and stored at −80 °C. A myrosinase unit was defined as the amount of enzyme required to hydrolyze 1 μmol of sinigrin per minute under conditions of pH 6.5 and 37 °C. Individual (IND) enzymatic digests of broccoli glucosinolates were performed with single compounds in the dose range of 0.5–50 μM for 2 h at 37 °C and neutral pH in the presence of 0.025 U myrosinase as described previously [37].

**Figure 2:**
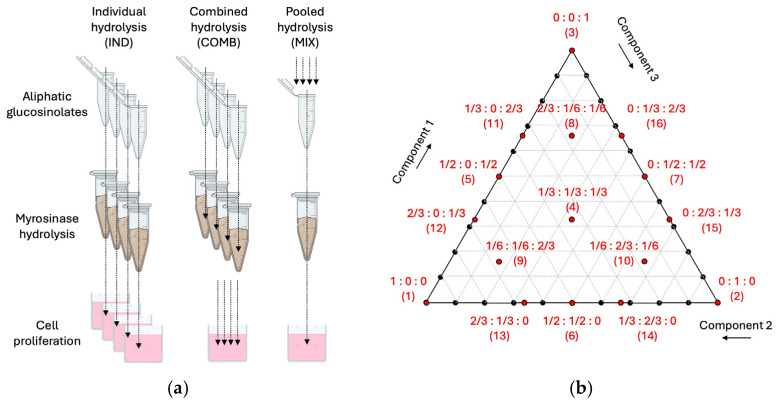
Schematic diagrams indicating the experimental setups to produce (**a**) individual hydrolysis (IND), combined hydrolysis (COMB), and pooled hydrolysis (MIX) of broccoli aliphatic glucosinolates in the presence of myrosinase, and (**b**) the augmented simplex-centroid mixture design model to evaluate glucosinolate–glucosinolate interactions at the level of myrosinase and their effect on the antiproliferative qualities in the human colorectal adenocarcinoma cells.

The effects of the myrosinase digestion of glucosinolate mixtures were evaluated under two separate conditions. Combined mixtures of individual glucosinolate digests (COMB) were performed by digesting each glucosinolate separately in the presence of myrosinase, then combining the digested glucosinolates in set proportions as specified in each experiment. Pooled digests of glucosinolates (MIX) were performed by pre-mixing the individual glucosinolates in the set proportions, then digesting the resulting mixture in the presence of myrosinase. The latter treatment allowed us to evaluate both positive and negative interactions among glucosinolates at the level of myrosinase digestion (Figure 2a).

Whole broccoli florets from the selected isogenic lines were solubilized at 200 mg/mL and subjected to enzymatic digestion in the presence of myrosinase similar to the pooled (MIX) glucosinolate digests.

### 2.4. Cell Culture

HT-29 human colorectal adenocarcinoma cells (ATCC HTB-38) were obtained from the American Type Culture Collection (Manasses, VA, USA). The cells were maintained in DMEM (Life Technologies, Carlsbad, CA, USA) supplemented with 10% fetal bovine serum, 100 IU/mL penicillin, and 100 μg/mL streptomycin (Fisher Scientific, Pittsburg, PA, USA) at a density not exceeding 5 × 10^5^ cells/mL. Passages were performed at 80–90% confluence every 3–4 days in 57 cm^2^ cell culture dishes (Nalge Nunc International, Rochester, NY, USA) maintained at 37 °C in a humidified 5% CO_2_ Thermo Forma Series II incubator (Fisher Scientific). All cell culture work was performed within the cell passages 2–16.

### 2.5. Antiproliferative Cell Assay

Changes in the proliferation of the HT-29 colonic cells in response to individual (IND), combined (COMB), and pooled mixtures (MIX) of broccoli glucosinolates were quantified using the a Sulforhodamine B (SRB) assay [38,39]. Briefly, the cells were seeded at a concentration of 1 × 10^4^ cells per well in 96-well plates with 100 μL of complete media. After a 4 h incubation, myrosinase hydrolysis treatments were administered in a 100 μL vehicle (0.7% DMSO in complete medium). Paclitaxel (Taxol), a natural anticancer cyclodecane isolated from the bark of the pacific yew tree *Taxus brevifolia* Nutt., was used as a reference positive control at 0.5 μM [40].

Following a 72 h incubation, the media was removed and living cells were fixed to plates with 10% trichloroacetic acid at 4 °C for 1 h. The plates were then washed 4 times with water to remove dead cells and dried overnight. The fixed cells were stained with 100 μL of 0.056% SRB dye (Sigma-Aldrich) in 1% acetic acid for 30 min. Dye solution was then discarded and the plates were washed with 1% acetic acid 4 times to remove any residual dye. The remaining SRB dye bound to cells was extracted with 200 μL of 10 mM Trizma and incubation with shaking for 30 min. Cell proliferation was determined as a percent reduction in absorbance values at 510 nm compared to the vehicle control using a BioTek Synergy H1 spectrophotometer (Winooski, VT, USA). All samples were tested in triplicate.

### 2.6. Determination of IC_50_ Values

IC_50_ values for individual (IND), combined (COMB), and pooled mixtures (MIX) of broccoli glucosinolates were calculated using a four-parameter nonlinear log(inhibitor) versus a proliferative response model in GraphPad Prism 6.0 (San Diego, CA, USA).

### 2.7. Determination of Combination Indexes (CI)

The combination index was calculated to determine the nature of interactions between individual glucosinolates in combined and pooled mixtures at various concentrations using the previously described formulas [34]. Briefly, the dual CIs were calculated as (d1/dx1 + d2/dx2) + (d1 × d2)/(dx1 × dx2) where d1 and d2 were the concentrations (IC50 values) of the compounds 1 and 2 that, in combination, give the same response as compound 1 (dx1) or compound 2 (dx2) alone. Likewise, triple CIs were calculated as (d1/dx1 + d2/dx2 + d3/dx3) + (d1 × d2 × d3)/(dx1 × dx2 × dx3). A CI of less than 1 indicated a synergistic interaction, a CI equal to 1 indicated an additive interaction, and a CI greater than 1 indicated an antagonistic interaction [34]. The resulting CIs were scored as very strong synergism (<0.1), strong synergism (0.1–0.3), synergism (0.3–0.7), moderate synergism (0.7–0.8), weak synergism (0.8–0.9), nearly additive (0.9–1.1), weak antagonism (1.1–1.2), moderate antagonism (1.2–1.5), antagonism (1.5–3.3), strong antagonism (3.3–10), and very strong antagonism (>10) [34,41].

### 2.8. Mixture Design Model

The augmented simplex-centroid mixture design method [42] was also applied to the combined (COMB) and pooled mixtures (MIX) of broccoli glucosinolates for an analysis of their antiproliferative effects. The method was selected to create a balanced design of 16 glucosinolate mixtures including 3 singular, 9 binary, and 4 tertiary interactions (Figure 2b and Table 1). The mixture analysis was performed at a dose level of 50 μM and visualized using JMP Pro 12.0 (SAS Institute, Cary, NC, USA) using a standard least squares model to create predictions and surface responses. The experiments were conducted in random order to break down any occurring systematic time trends. Interactions between each variable were investigated for significance using an alpha of 0.01 and corrected for false discovery rate (FDR) and parameter estimate. These data were used to determine the optimal ratios of aliphatic glucosinolates and predict an enhanced broccoli glucosinolate profile to maximize its antiproliferative effects.

### 2.9. Statistical Analysis

Statistical analysis was performed using Prism 6.0 (GraphPad) and expressed as means ± SEM. Two tailed *t*-test or one-way ANOVA were applied at a significance level of *p* < 0.05. Post hoc analyses of differences between individual experimental groups were made using Tukey’s multiple comparison test. Combination indexes and mixture analysis were performed as described above.

## 3. Results

### 3.1. Comparative Antiproliferative Potencies of Aliphatic Glucosinolates

**Figure 3:**
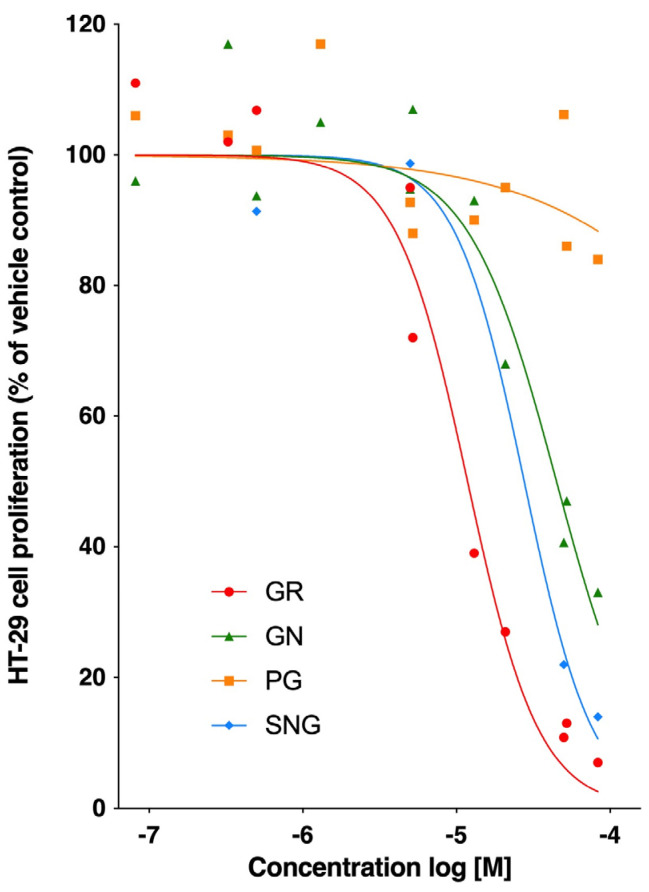
Antiproliferative effects of aliphatic glucosinolates from broccoli. HT-29 human colon cancer cells were treated with 0.5–50 μM myrosinase-hydrolyzed glucosinolates to establish the corresponding IC_50_ values for individual compounds.

Intact broccoli aliphatic glucosinolates (sinigrin, glucoraphanin, gluconapin, progoitrin) in their native form were evaluated individually for their antiproliferative effects against the HT-29 human colorectal adenocarcinoma cells in the dose range of 0.5–50 μM and were observed to be inactive in line with the previous reports [15]. Myrosinase-catalyzed hydrolysis of target aliphatic glucosinolates was then performed in the individual tubes (IND) before adding cells under the same treatment conditions (Figure 2a). Individual myrosinase hydrolysis showed significant antiproliferative effects in HT-29 cells in the order of potency glucoraphanin (IC_50_ = 11.80 µM) > sinigrin (IC_50_ = 29.51 µM) > gluconapin (IC_50_ = 44.76 µM) > progoitrin (IC_50_ = 2100.6 µM) (Figure 3). Since aliphatic glucosinolates are a group of closely related compounds that share a β-thioglucose and a sulfonated oxime moiety, but differ at the variable aglycone side chain, we next evaluated the direct glucosinolate–glucosinolate interactions and their antiproliferative potential at the level of myrosinase that is critical for their activation.

### 3.2. Combination Index of Aliphatic Glucosinolates

Two mixing strategies were evaluated to understand glucosinolate–glucosinolate interactions—the combined hydrolysis (COMB) that included individual glucosinolates hydrolyzed in the presence of the enzyme (no competition at the level of myrosinase) and the pooled mixes (MIX) that included the target glucosinolate premixes that compete at the active site of myrosinase for their processing and hydrolysis (Figure 2a). The combination index of aliphatic glucosinolates was calculated at the IC_50_ concentration for all compounds in COMB and MIX mixtures (Table 1). A pooled MIX mixture that included only gluconapin, progoitrin, and sinigrin showed weak antiproliferative activity (>2500 µM) that did not reach 50% inhibition of cell proliferation relative to the vehicle control. The combination index could not be calculated for this sample.

The inclusion of additional aliphatic glucosinolates into reactions with glucoraphanin decreased the resulting IC_50_ antiproliferative values of the resulting mixtures at the level of the colonic cells (COMB) as well at the level of myrosinase (MIX). The effect was particularly evident at the level of myrosinase (MIX) with an average 4- to 6-fold decrease in the IC_50_ proliferative activity (Table 1). The conventional combination indexes calculated based on the IC_50_ values indicated moderate to full antagonism for the majority of the mixtures (CIs in the range of 1.2–2.9). Progoitrin in combination with glucoraphanin and gluconapin showed the strongest antagonistic effect at the level of cells (3.88), while the other aliphatic glucosinolates showed a moderate antagonistic effect to glucoraphanin at the level of myrosinase (2.00 and 2.93, respectively).

Taken together, the antagonism was higher in pooled hydrolysis (MIX) rather than combined hydrolysis (COMB), suggesting a primary negative glucosinolate–glucosinolate interaction at the level of myrosinase. The antagonistic effect was nearly absent in the mixtures that did not include glucoraphanin (such as equal amounts of gluconapin, progoitrin, and sinigrin), indicating that glucoraphanin was the major glucosinolate responsible for the observed antiproliferative effects (Table 1, line 6). Glucoraphanin and gluconapin showed a moderate additive interaction independent of myrosinase (Table 1, line 12).

### 3.3. Mixture Design Analysis for Nutrient Interactions

The conventional IC_50_ and combination index analyses indicated a complex variety of antagonistic and additive interactions among the aliphatic glucosinolates in broccoli. We next employed mixture design analysis commonly applied in food science to evaluate food ingredient functionality or their organoleptic properties to generate a subset of experiments comprising the tetrahedral (four component) optimization model. The augmented simplex-centroid mixture design proportions and reference names for glucosinolate mixtures are listed in Table 2.

**Figure 4:**
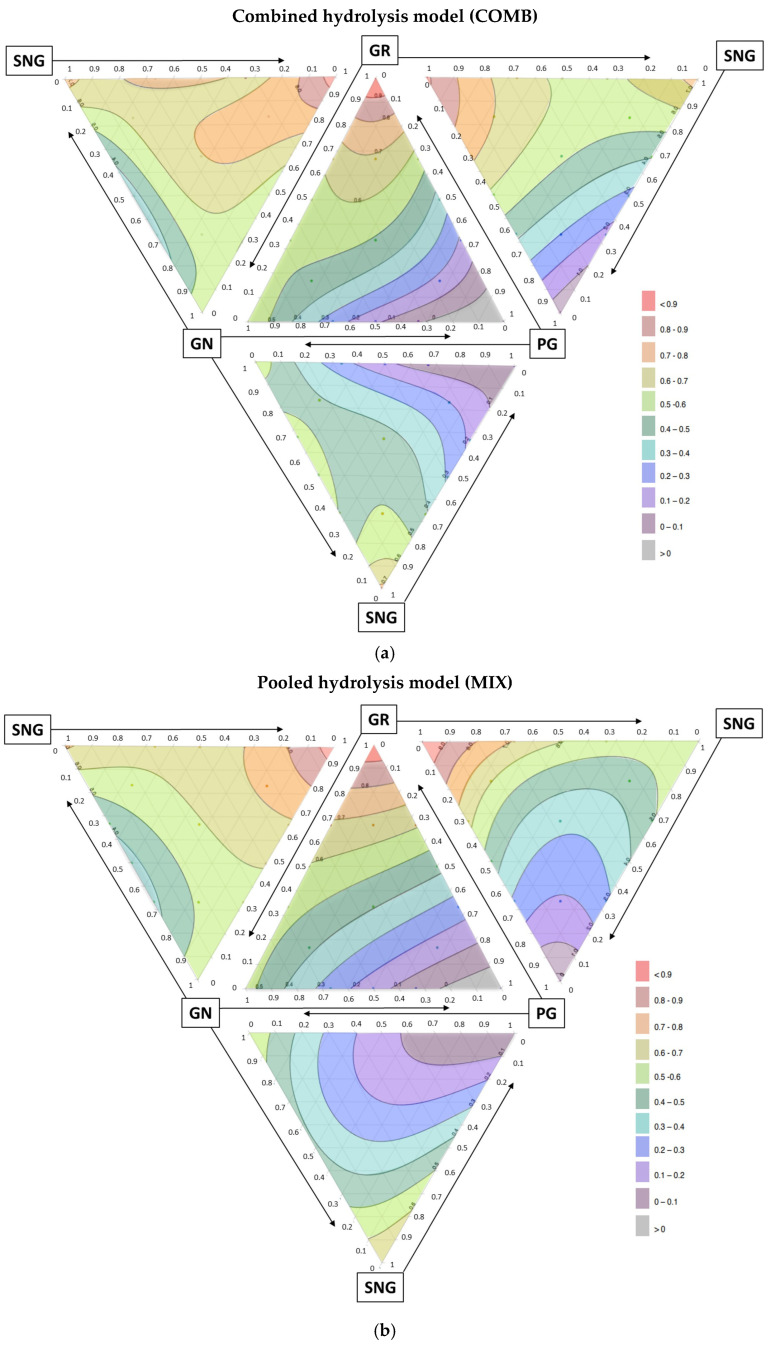
Mixture contour plots using a tetrahedral projection of (**a**) combined COMB and (**b**) pooled MIX hydrolysis of broccoli glucosinolates tested at 50 μM. The potency of antiproliferative effects against HT-29 cells is shown as a color gradient from gray (lowest) to red (highest). Arrows indicate the direction of the decreasing proportion for each individual glucosinolate in the mixture. Lateral panels were inverted to maintain the correct tetrahedral projections.

The glucosinolate–glucosinolate interactions were evaluated at 50 µM based on the average IC_50_ values obtained from the individual hydrolysis (Figure 4). All models were evaluated for goodness of fit (R^2^) and analysis of variance (ANOVA) to establish the significance of each interaction.

### 3.4. Significant Interactions Among Aliphatic Glucosinolates

The GR × GN × PG model (panel 1) showed that interactions between GR × GN (*p* = 0.01), as well as glucoraphanin and gluconapin proportions interacting with the total concentration of glucosinolates (*p* < 0.0001), were the most significant positive effects for variables contributing to inhibition of HT-29 cell proliferation in both COMB (R^2^ adj = 0.924) and MIX (R^2^ adj = 0.934) modes. On the other hand, the model predicted the most significant negative interaction between GR × GN × PG that increased at the level of myrosinase when these glucosinolates were tested in the pooled MIX model (*p* < 0.0001). These interactions could be easily identified on the mixture contour plots by the zone of the highest antiproliferative activity (red) observed at high glucoraphanin proportions; a zone of high antiproliferative activity (orange) expanding from glucoraphanin in the direction of gluconapin; and a vast zone of low antiproliferative activity (gray, purple, blue) found at high progoitrin proportions (Figure 4).

The GR × GN × SNG model (panel 2) showed the highest R^2^ adj values in both COMB (R^2^ adj = 0.948) and MIX (R^2^ adj = 0.967) modes, indicating that the removal of progoitrin from the mixture resulted in increased antiproliferative activity. In the COMB mode, all interactions besides GR × GN proportion × concentration were significantly associated with bioactivity (*p* < 0.01). In the MIX mode, GR × GN × SNG proportion was a non-significant factor compared to being highly significant to the COMP mode (*p* < 0.0001), once again suggesting a direct competition among the individual glucosinolates at the level of myrosinase.

The GR × PG × SNG model (panel 3) showed the lowest R^2^ adj of all mixtures in both COMB (R^2^ adj = 0.870) and MIX (R^2^ adj = 0.806) modes. This model found only one significant association with the inhibition of HT-29 cell proliferation between GR × SNG (*p* < 0.01). When glucoraphanin was excluded from the mixtures, the GN × PG × SNG model (panel 4) in both the COMB (R^2^ adj = 0.894) and MIX (R^2^ adj = 0.855) modes indicated new negative associations between GN × PG in both the COMB (*p* < 0.0001) and MIX (*p* < 0.02) modes, as well as a negative association between SNG × PG in the COMB mode only (*p* = 0.02).

Taken together, the mixture design analysis predicted that an 80% threshold inhibition of the proliferation of HT-29 human colon cancer cells could be achieved at a 50 µM dose by the following ratio of aliphatic glucosinolates: 81–84% glucoraphanin, 9–19% gluconapin, and 0–7% others.

### 3.5. Predicted Optimal Aliphatic Glucosinolate Ratio in Broccoli Population

**Figure 5:**
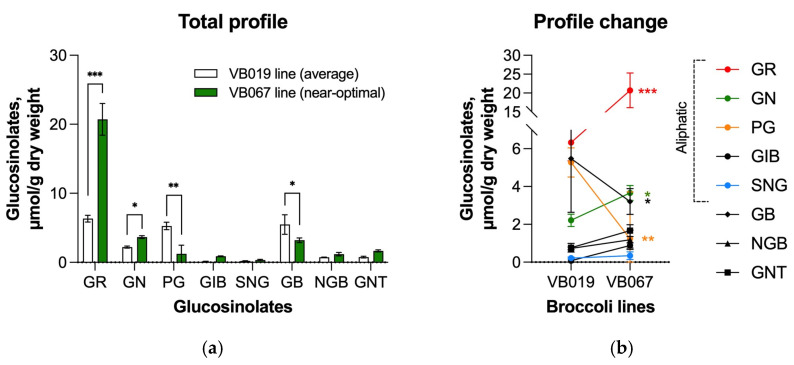
Total glucosinolate profile (**a**) and the profile change (**b**) of two selected broccoli isogenic lines with an average (VB019) and a near-optimal (VB067) profile identified in this study. All glucosinolates including aliphatic glucoraphanin (GR), gluconapin (GN), progoitrin (PG), glucoiberin (GIB), sinigrin (SGN), aromatic glucobrassicin (GB), neoglucobrassicin (NGB), and indolic gluconasturitiin (GNT) were quantified by HPLC. The data were analyzed using one-way ANOVA followed by Dunnett’s multiple comparisons, * *p* < 0.05, ** *p* < 0.01, *** *p* < 0.001.

The F2:3 broccoli mapping population described earlier [30] was next screened for the isogenic broccoli lines that exhibited the desired ratio. The VB067 isogenic line exhibited a near-optimal aliphatic glucosinolate profile in the form of 77.2% glucoraphanin, 13.6% gluconapin, 4.6% progoitrin, 3.3% glucoiberin, and 1.3% sinigrin. The rest of the mapping population was evaluated to identify an average (most common) aliphatic glucosinolate profile. The sister VB019 isogenic line exhibited the closest average aliphatic glucosinolate profile in the form of 54.9% glucoraphanin, 19.2% gluconapin, 23.4% progoitrin, 0.7% glucoiberin, and 1.9% sinigrin (Figure 5a). The relative changes in individual glucosinolate amounts between these lines are highlighted in Figure 5b.

### 3.6. Antiproliferative Activity of the Selected Broccoli Isogenic Lines

**Figure 6:**
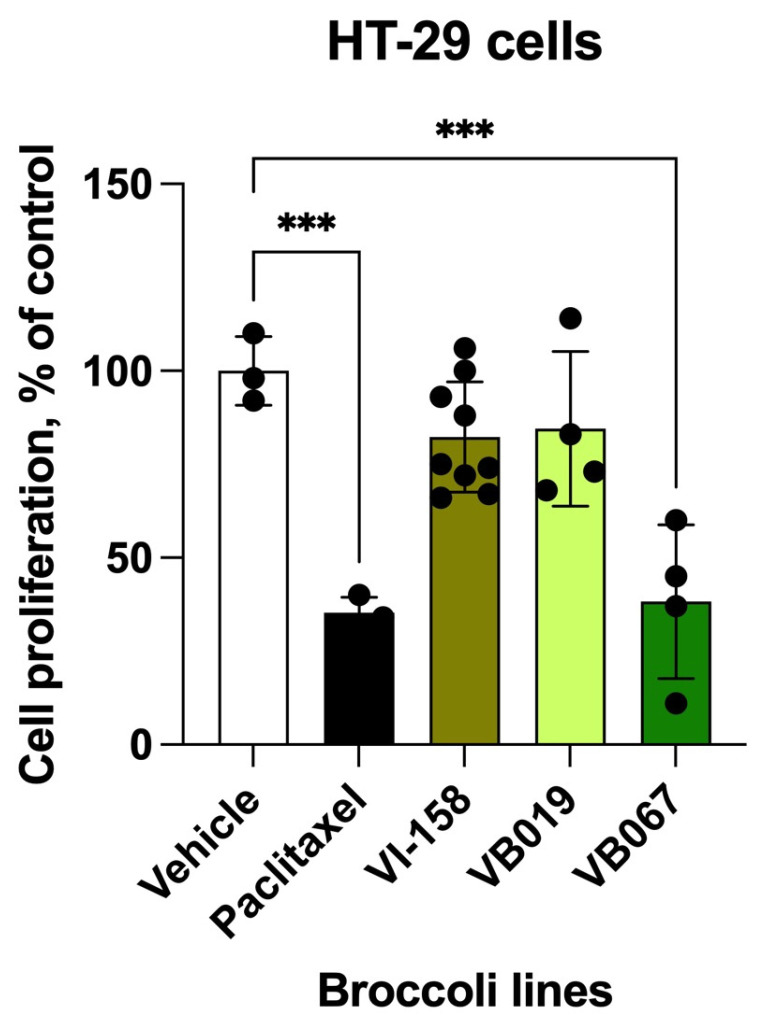
Antiproliferative effects of two selected broccoli isogenic lines with an average (VB019) and near-optimal (VB067) profile identified in this study as compared to the commercial parent line (VI-158) and a reference drug (paclitaxel at 10 µM). HT-29 human colon cancer cells were treated with myrosinase-hydrolyzed whole broccoli floret extracts. The data were analyzed using one-way ANOVA followed by Dunnett’s multiple comparisons, *** *p* < 0.001.

The two broccoli isogenic lines VB019 and VB067 were then compared for their ability to inhibit HT-29 human colon cancer cell growth together with the commercial parent broccoli cultivar (VI-158), and a reference drug (paclitaxel at 10 µM). A typical aliphatic glucosinolate profile of commercial broccoli contains 47% glucoraphanin, 4% gluconapin, 42% progoitrin, and 1% sinigrin [24]. The near-optimal VB067 broccoli line showed a 44.1% increase in antiproliferative activity over its commercial parent VI-158, and a 46.3% increase over its average sister VB019 broccoli line (Figure 6).

## 4. Discussion

The recommended way for individuals to obtain health-beneficial phytochemicals is through food sources, as the combined effects of different metabolites in whole foods may enhance their desired health outcomes. Classical examples of food–food interactions that support this concept are increased non-heme iron absorption from spinach after its reduction by vitamin C found in orange juice [43], the enhanced bioavailability of carrot beta-carotene when consumed with dietary oils [44], and greater intake of curcumin when turmeric is consumed with black pepper that contains piperine [45].

Traditionally, entire phytochemical groups such as polyphenols, glucosinolates, and carotenoids were evaluated for nutrient–nutrient and nutrient–food matrix interactions based on their shared chemical structures and general biological activities [4], and this message is further simplified for the general public [46]. Their collective benefits, however, remain poorly understood due to the limited knowledge of how closely related nutrients or phytochemicals within a single phytochemical group interact with each other. These compounds often share a similar core structure but differ in specific structural features, such as functional groups, side chains, or degrees of glycosylation that influence their bioavailability, metabolism, and bioactivity in the body [47].

For instance, glucosinolates, which are prominent in cruciferous vegetables, differ in side-chain length and composition, influencing their hydrolysis rates and the bioactivity of their resulting isothiocyanates [48,49]. While glucoraphanin and its breakdown isothiocyanate sulforaphane have been widely studied for their chemopreventive properties [50], understanding glucosinolate–glucosinolate interactions is essential because the bioactivity of a single compound may not fully represent the combined effects of a phytochemical group. Broccoli contains eight major glucosinolates, with total concentrations ranging from 8.4 to 19.5 µmol/g fresh weight in commercial varieties [24]. Without a clear understanding of how these glucosinolates interact, their combined health benefits cannot be accurately assessed or optimized for dietary recommendations or breeding programs focused on enhancing phytochemical profiles.

In this study, our approach aimed to identify positive and negative interactions of major aliphatic glucosinolates in broccoli (glucoraphanin, gluconapin, progoitrin, and sinigrin) that contribute to the overall bioactivity of the crop. While classical methods, such as IC_50_ isobolograms and combination index analysis [34], are typically used to evaluate the combined effects of phytochemicals, they often fall short in capturing the complexity of interactions within the food matrix and predicting the optimal range of bioactive constituents. Alternatively, mixture analysis, a statistical model-based method commonly used in sensory optimization to assess product functionality, desirability, and consumer acceptance, offers a robust framework for characterizing complex interactions and identifying the optimal proportions of the individual components [51].

Our data confirmed glucoraphanin to be the primary contributor to antiproliferative activity in a mixture of aliphatic glucosinolates as suggested previously [52], with much lesser contributions from gluconapin and sinigrin. Progoitrin alone exhibited negligible ability to inhibit cell growth. Classical combination analyses then revealed moderate to strong antagonistic interactions between glucoraphanin and other glucosinolates at the level of myrosinase, except for gluconapin, though the low bioactivity of progoitrin made its contribution difficult to quantify.

This limitation was addressed using mixture analysis [42]. Surface response mixture models predicted the optimal ratio of aliphatic glucosinolates for maximum antiproliferative activity to be 81–84% glucoraphanin, 9–19% gluconapin, and 0–7% other glucosinolates. This combination achieved a 61.8% inhibition threshold of HT-29 human colon cancer cell proliferation at a 50 µM dose. Plasma concentrations of sulphoraphane, a major enzymatic breakdown metabolite of glucoraphanin, reach 15–20 µM in humans, putting our findings in the range of physiologically relevant bioactivity [53]. Direct comparisons between the antiproliferative bioactivity of individually hydrolyzed glucosinolates (COMB) and pooled hydrolysis (MIX) revealed mostly negative associations with other glucosinolates, and especially progoitrin. These results strongly suggest that competition for enzymatic hydrolysis by myrosinase reduced individual glucosinolate contributions to bioactivity. We expect this to be significant not only at the level of broccoli tissue myrosinases [54], but also with microbial myrosinases found within the gut microbiome [55]. In a previous study, the induction of phase II detoxification enzymes at low isothiocyanate levels was shown as another independent driver of antagonism between glucoraphanin and other glucosinolates [56]. The gut microbiota plays an additional role in modulating the antiproliferative effects of glucosinolates by producing microbial myrosinases that hydrolyze these compounds into bioactive isothiocyanates [55]. This enzymatic hydrolysis is critical for activating glucoraphanin into sulforaphane, a potent inducer of phase II detoxification enzymes that reduces oxidative stress and inhibits cancer cell proliferation, and angiogenesis, as well as conferring epigenetic effects [57]. These interactions underscore the complex interplay between dietary phytochemicals and gut microbiota in shaping their antiproliferative potential, emphasizing the need to account for individual microbiota variability in future evaluations of functional foods like broccoli.

This approach was validated as an independent investigation arm study within the previously developed broccoli breeding program [30]. Testing the near-optimal VB067 isogenic broccoli line revealed a 44% increase in antiproliferative activity compared to the commercial parent and an average breeding sister line, further supporting the significance of optimizing glucosinolate profiles for enhanced bioactivity. These findings also have a broader significance in understanding the health benefits of other structurally related phytochemical groups, where interactions between metabolites can enhance or become detrimental to their desired biological effects, i.e., phenolics [58].

One limitation of this study is the exclusion of other classes of glucosinolates from the analysis (unavailable at the time in quantities sufficient to perform the required cell culture work). For example, the hydrolysis products of glucobrassicin and neoglucobrassicin (indole-3-carbinols) are known to exhibit antiproliferative activity but are also associated with contributing to the off-flavors of *Brassica* vegetables [59]. Their potential interactions with the hydrolysis and bioactivity of aliphatic glucosinolates in broccoli deserve further investigation. Expanding the scope of this study to include additional factors relevant to chemoprevention could provide a deeper mechanistic understanding of how these compounds interact synergistically or antagonistically. Finally, breeding broccoli with the optimal glucosinolate profile alone does not guarantee enhanced health benefits for consumers. Post-harvest handling, processing, and cooking methods can inactivate myrosinase or promote the formation of alternative breakdown metabolites, potentially altering the intended bioactivity [60].

## 5. Conclusions

The results of this study underscore the fact that treating phytochemicals as homogeneous groups with similar biological activities oversimplifies their interactions and potential health impacts, posing a significant challenge in nutritional science and public health communication. By analyzing the complex interactions among closely related glucosinolates in broccoli, we demonstrated how mixture analysis can identify the optimal proportions of these compounds to maximize biological activity, even when individual bioactivity is too low to produce IC_50_ values. Our findings strongly support the molecular breeding of broccoli with increased glucoraphanin levels and reduced proportions of antagonistic glucosinolates, especially progoitrin, as the competition for enzymatic hydrolysis by myrosinase plays a crucial role in modulating the overall bioactivity of these compounds. Ultimately, these insights will inform future breeding and dietary recommendations aimed at enhancing the antiproliferative properties of cruciferous vegetables.

## Figures and Tables

**Table 1:** Interactions among broccoli aliphatic glucosinolates in their ability to suppress proliferation of HT-29 human colorectal adenocarcinoma cells, estimated by conventional IC_50_ and CI values.

Interactions		Proportion Ratios				IC_50_ Value			Combination Index	
		GR	GN	PG	SNG	IND	COMB	MIX	COMB	MIX
1	Singular	1	–	–	–	11.80	–	–	–	–
2	Singular	–	1	–	–	44.76	–	–	–	–
3	Singular	–	–	1	–	2100.62	–	–	–	–
4	Singular	–	–	–	1	29.51	–	–	–	–
5	Tertiary	1/3	1/3	1/3	–	–	64.19	52.25	2.31	1.88
6	Tertiary	–	1/3	1/3	1/3	–	54.23	62.06	1.03	1.18
7	Tertiary	1/3	–	1/3	1/3	–	28.76	50.16	1.41	2.66
8	Tertiary	1/3	1/3	–	1/3	–	27.69	46.18	1.35	2.40
9	Tertiary	2/3	1/6	1/6	–	–	24.66	45.24	1.49	2.73
10	Tertiary	2/3	–	1/6	1/6	–	25.94	35.03	1.61	2.18
11	Tertiary	2/3	1/6	–	1/6	–	29.84	43.07	2.00	2.93
12	Tertiary	1/6	2/3	1/6	–	–	48.01	56.32	0.99	1.16
13	Tertiary	1/6	2/3	–	1/6	–	47.74	50.23	1.78	1.89
14	Tertiary	1/6	1/6	2/3	–	–	205.83	62.74	3.88	1.14
15	Tertiary	1/6	1/6	–	2/3	–	48.18	50.00	2.08	2.17
16	Tertiary	1/6	–	2/3	1/6	–	58.31	59.65	1.18	1.20
17	Tertiary	1/6	–	1/6	2/3	–	47.09	48.24	1.73	1.78
18	Tertiary	–	2/3	1/6	1/6	–	66.16	62.67	1.37	1.29
19	Tertiary	–	1/6	2/3	1/6	–	57.15	>2,500	0.55	nd
20	Tertiary	–	1/6	1/6	2/3	–	47.82	51.77	1.26	1.37

Abbreviations: (–) not present in mixture; (nd) not determined.

**Table 2:** Mixture design analysis model performed in combined (COMB) and pooled (MIX) hydrolysis.

Interactions		Panel 1			Panel 2			Panel 3			Panel 4		
		GR	GN	PG	GR	GN	SNG	GR	PG	SNG	GN	PG	SNG
1	Singular	1	–	–	1	–	–	1	–	–	1	–	–
2	Singular	–	1	–	–	1	–	–	1	–	–	1	–
3	Singular	–	–	1	–	–	1	–	–	1	–	–	1
4	Tertiary	1/3	1/3	1/3	1/3	1/3	1/3	1/3	1/3	1/3	1/3	1/3	1/3
5	Binary	1/2	–	1/2	1/2	–	1/2	1/2	–	1/2	1/2	–	1/2
6	Binary	1/2	1/2	–	1/2	1/2	–	1/2	1/2	–	1/2	1/2	–
7	Binary	–	1/2	1/2	–	1/2	1/2	–	1/2	1/2	–	1/2	1/2
8	Tertiary	2/3	1/6	1/6	2/3	1/6	1/6	2/3	1/6	1/6	2/3	1/6	1/6
9	Tertiary	1/6	1/6	2/3	1/6	1/6	2/3	1/6	1/6	2/3	1/6	1/6	2/3
10	Tertiary	1/6	2/3	1.6	1/6	2/3	1.6	1/6	2/3	1.6	1/6	2/3	1.6
11	Binary	1/3	–	2/3	1/3	–	2/3	1/3	–	2/3	1/3	–	2/3
12	Binary	2/3	–	1/3	2/3	–	1/3	2/3	–	1/3	2/3	–	1/3
13	Binary	2/3	1/3	–	2/3	1/3	–	2/3	1/3	–	2/3	1/3	–
14	Binary	1/3	2/3	–	1/3	2/3	–	1/3	2/3	–	1/3	2/3	–
15	Binary	–	2/3	1/3	–	2/3	1/3	–	2/3	1/3	–	2/3	1/3
16	Binary	–	1/3	2/3	–	1/3	2/3	–	1/3	2/3	–	1/3	2/3

Abbreviations: (–) not present in the mixture.

## Data Availability

The original contributions presented in this study are included in the article. Further inquiries can be directed to the corresponding author.
